# *Ecklonia Cava* Extract Attenuates Endothelial Cell Dysfunction by Modulation of Inflammation and Brown Adipocyte Function in Perivascular Fat Tissue

**DOI:** 10.3390/nu11112795

**Published:** 2019-11-15

**Authors:** Myeongjoo Son, Seyeon Oh, Hye Sun Lee, Dong-Min Chung, Ji Tae Jang, You-Jin Jeon, Chang Hu Choi, Kook Yang Park, Kuk Hui Son, Kyunghee Byun

**Affiliations:** 1 Department of Anatomy & Cell Biology, Gachon University College of Medicine, Incheon 21936, Korea; mjson@gachon.ac.kr; 2 Functional Cellular Networks Laboratory, College of Medicine, Department of Medicine, Graduate School and Lee Gil Ya Cancer and Diabetes Institute, Gachon University, Incheon 21999, Korea; seyeon8965@gachon.ac.kr (S.O.); hsl@gachon.ac.kr (H.S.L.); 3 Shinwoo cooperation. Ltd. 991, Worasan-ro, Munsan-eup, Jinju, Gyeongsangnam-do 52839, Korea; jdm@shinwoocorp.com; 4 Aqua Green Technology Co., Ltd., Smart Bldg., Jeju Science Park, Cheomdan-ro, Jeju 63309, Korea; whiteyasi@gmail.com; 5 Department of Marine Life Science, Jeju National University, Jeju 63243, Korea; youjin2014@gmail.com; 6 Department of Thoracic and Cardiovascular Surgery, Gachon University Gil Medical Center, Gachon University, Incheon 21565, Korea; cch624@gilhospital.com (C.H.C.); kkyypark@gilhospital.com (K.Y.P.)

**Keywords:** obesity, *Ecklonia cava*, perivascular fat tissue, brown adipocyte, endothelial cell dysfunction, endoplasmic reticulum stress, inflammation

## Abstract

It is well known that perivascular fat tissue (PVAT) dysfunction can induce endothelial cell (EC) dysfunction, an event which is related with various cardiovascular diseases. In this study, we evaluated whether *Ecklonia cava* extract (ECE) and pyrogallol-phloroglucinol-6,6-bieckol (PPB), one component of ECE, could attenuate EC dysfunction by modulating diet-induced PVAT dysfunction mediated by inflammation and ER stress. A high fat diet (HFD) led to an increase in the number and size of white adipocytes in PVAT; PPB and ECE attenuated those increases. Additionally, ECE and PPB attenuated: (i) an increase in the number of M1 macrophages and the expression level of monocyte chemoattractant protein-1 (MCP-1), both of which are related to increases in macrophage infiltration and induction of inflammation in PVAT, and (ii) the expression of pro-inflammatory cytokines (e.g., tumor necrosis factor-α (TNF-α) and interleukin (IL)-6, chemerin) in PVAT which led to vasoconstriction. Furthermore, ECE and PPB: (i) enhanced the expression of adiponectin and IL-10 which had anti-inflammatory and vasodilator effects, (ii) decreased HFD-induced endoplasmic reticulum (ER) stress and (iii) attenuated the ER stress mediated reduction in sirtuin type 1 (Sirt1) and peroxisome proliferator-activated receptor γ (PPARγ) expression. Protective effects against decreased Sirt1 and PPARγ expression led to the restoration of uncoupling protein -1 (UCP-1) expression and the browning process in PVAT. PPB or ECE attenuated endothelial dysfunction by enhancing the pAMPK-PI3K-peNOS pathway and reducing the expression of endothelin-1 (ET-1). In conclusion, PPB and ECE attenuated PVAT dysfunction and subsequent endothelial dysfunction by: (i) decreasing inflammation and ER stress, and (ii) modulating brown adipocyte function.

## 1. Introduction

White adipose tissue (WAT), located in subcutaneous or intravisceral regions, is a primary energy store; brown adipose tissue (BAT) occupies the clavicular, cervical, suprarenal, and periaortic regions, generates heat, and functions in energy expenditure [1,2]. Most large blood vessels are covered by a specialized adipose tissue called perivascular adipose tissue (PVAT) [3]. Depending on the location, the composition of adipose tissue and function of PVAT is different [4]. The PVAT around the thoracic aorta shares features similar to BAT including: (i) adipocytes with multiple lipid droplets and high mitochondrial content, and (ii) high relative expression of thermogenic genes like uncoupling protein-1 (UCP-1) [5]. However, PVAT around the abdominal aorta is more similar to WAT, namely: (i) adipocytes with large lipid droplets and low mitochondrial content, and (ii) low expression of UCP-1 [6]. PVAT has been proven essential for modulating vascular function through the production and release of autocrine and paracrine signaling molecules [7,8]. These molecules are vasoactivators and function as either vasorelaxants or vasoconstrictors [9,10]. Simple molecules (e.g., nitric oxide (NO), hydrogen sulfide), reactive oxygen species (e.g., hydrogen peroxide), and adipokines (e.g., adiponectin, angiotensin 1-7) are vasorelaxants from PAVT [11,12]. Tumor necrosis factor-α (TNF-α) and interleukin (IL)-6 enhance vasocontractions by upregulating endothelin signaling or reducing NO production [13,14,15].

In healthy PVAT, the secretory profile of paracrine and endocrine signaling molecules maintains the balance between vasodilation, anticontractile, and antiproliferative actions [16]. In addition, healthy PVAT serves an anti-inflammatory function by: (i) increasing the secretion of anti-inflammatory cytokines (e.g., IL-10), and (ii) minimizing the effects of adiponectin on TNF-α and C-reactive protein [17,18]. Importantly, obesity induces an increase in the size and number of white adipocytes in PVAT [19,20], and these morphological or structural changes are accompanied by dysfunction (e.g., abnormal adipokine production, oxidative stress, hypoxia, inflammation in the adipose tissue) [21]. Obesity-induced PVAT hypertrophy causes hypoperfusion and local hypoxia because the diffusion of oxygen is hindered by the size of adipocytes [22,23]. Hypoxia in adipose tissue induces an increase in the expression of the chemokine monocyte chemoattractant protein-1 (MCP-1), which promotes recruitment and infiltration of macrophages and the secretion of pro-inflammatory cytokines such as TNF-α from macrophages [19,24]. A high fat diet (HFD) leads to an increase in pro-inflammatory adipokines/cytokines (e.g., leptin, IL-6, TNF-α, and interferon-γ) and decrease in anti-inflammatory adipokines/cytokines (e.g., adiponectin and IL-10) in PVAT [25,26]. This imbalance aggravates inflammation and promotes PVAT dysfunction, leading to subsequent dysregulation of vasoactivators in PVAT [8,27]. PVAT dysfunction induces an upregulation of vasoconstricting factors [8] and is related with obesity and hypertension [23,28]. In addition, PVAT dysfunction contributes to endothelial cell (EC) dysfunction induced by obesity [19] and vascular smooth muscle cell (VSMC) dysfunction [28]. Thus, studies have clearly shown that PVAT dysfunction is related with EC and VSMC dysfunction which may lead to cardiovascular diseases, including hypertension, atherosclerosis, and coronary heart diseases [11,29]. Based on studies which have shown that PVAT dysfunction is a causative factor of cardiovascular disease, several studies showed that an increased proportion of brown-to-white adipocytes improved vascular function in individuals suffering from obesity and atherosclerosis [30,31].

Cellular stress induced by obesity increases the production of unfolded proteins, an event which enhances endoplasmic reticulum (ER) stress and subsequent aggravation of inflammation and induction of cell death [32]. HFD-induced ER stress [33] in PVAT is mediated by the upregulation of pro-inflammatory cytokines such as IL-1β and IL-6 and decreases in the abundance of protective adipokines such as adiponectin [34]. Those changes in PVAT ultimately induce EC dysfunction [34]. In addition, it is known that ER stress inhibits sirtuin type 1 (Sirt1) expression and damaged UCP-1 function in BAT [35]. Sirt1 improves whole-body energy expenditure by decreasing fat accumulation in white adipocytes [36,37], and increases metabolic activity by promoting the brown remodeling of white adipocytes [38,39,40].

The phlorotannins from *Ecklonia cava* (*E. cava*), is an edible brown seaweed and is found on coastal shore near Jeju, Korea have multiple biological activities, including anti-inflammatory [41,42], antioxidant [43], and anti-adipogenic [44]. In addition, *E. cava*-sourced phlorotannins have been reported to prevent EC death [45,46]. In previous studies, our group compared four representative phlorotannins (dieckol, 2,7-phloroglucinol-6,6-bieckol, Pyrogallol-phloroglucinol-6,6-bieckol and phlorofucofuroeckol A) from *E. cava* extract (ECE) and reported that Pyrogallol-phloroglucinol-6,6-bieckol (PPB): (i) inhibited monocyte-induced EC death by upregulating the phosphorylation of PI3K-AKT and AMPK, and (ii) inhibited monocyte-associated VSMC proliferation [45]. In addition, our group reported that PPB decreased adhesion molecule expression, EC death, and the proliferation of VSMCs in vitro and in mouse models of obesity and hypertension [45]. Although ECE, and PPB specifically have been shown to protect EC from monocyte-induced inflammation and EC death, there are no studies which have evaluated the effect of ECE on PVAT dysfunction and vascular dysfunction induced by obesity. Here, we evaluated whether ECE and PPB could attenuate inflammation and ER stress in PVAT, thus leading to a reduction in PVAT dysfunction and subsequent EC dysfunction in the diet-induced obesity (DIO) animal model.

## 2. Materials and Methods

### 2.1. DIO Animal Model

Male C57BL/6N mice (8 weeks of age) were obtained from Orient bio (Seongnam, Korea) and kept at a constant temperature of roughly 23 °C, relative humidity of 50% and a dark/light cycle of 12/12 hrs. Mice were fed different diets as described below and provided drinking water ad libitum for eight weeks. For the first four weeks, mice received either a regular chow diet (control), or a 45% high fat diet (research diet, USA) adapted from a previous study [45]. Diet-induced obesity model (DIO model) used to study obesity using mice that have obesity caused by being fed high fat diets.

For the last four weeks, DIO mice were orally administered 0.9% normal saline (Control or DIO/Saline), *E. cava* extract (DIO/ECE; 70 mg/kg/day) or Pyrogallol-phloroglucinol-6,6’-bieckol; PPB (DIO/PPB; 2.5 mg/kg/day) along with either a regular chow diet (control) or DIO. ECE and PPB doses used here were the same as a previous study [45]. At the end of the eight-week study period, all mice were sacrificed in accordance with the Ethical Principles in Institutional Animal Care and Use Committee of Gachon University (approval number; LCDI-2017-0034).

### 2.2. Preparation of E. cava Extract and Isolation of PPB

*E. cava* was obtained from Aqua Green Technology Co., Ltd. (Jeju, Korea). For extraction, *E. cava* were washed and air-dried at room temperature for 48 hrs, the leaves were ground, and 50% ethanol was added followed by incubation at 85 °C for 12 hrs. The *E. cava* extracts (ECE) were filtered, concentrated, sterilized by heating to over 85 °C for 40–60 min and then spray-dried. PPB was isolated following a previously reported procedure [46,47]. Simply, centrifugal partition chromatography (CPC) was performed using a two-phase solvent system comprised of water/ethyl acetate/methyl alcohol/n-hexane (7:7:3:2, v/v/v/v). The organic stationary phase was filled in the CPC column followed by pumping of the mobile phase into the column in descending mode at the same flow rate used for separation (2 mL per min). We finally confirmed the purity of the PPB is 91.24% was used in the study [46].

### 2.3. Immunohistochemistry (Immunofluorescence)

Blocks of paraffin-embedded aorta tissue were sectioned to a thickness of 7 µm, placed on a coating slide and dried at 40 °C for 24 hrs. Slides were deparaffinized, treated with normal animal serum to block non-specific binding, incubated with antibodies (ET-1 (abcam; UK, dilution rate 1:200), peNOS (abcam; dilution rate 1:250), UCP-1 (Santa Cruz Biotechnology, Inc.; dilution rate 1:200), Sirt1 (Santa Cruz Biotechnology, Inc.; dilution rate 1:200)) for two days at 4 °C, and then rinsed three times with PBS. Slides were then incubated for 1 h with Alexa Fluor Plus secondary antibodies (Thermo Fisher Scientific, MA, USA, dilution rate 1:500) and rinsed three times with PBS. Next, slides were incubated for 5 min with 4′ 6-diamidino-2-phenylindole (DAPI, Sigma-Aldrich) solution and then rinsed three times with PBS. Finally, cover slips and shield solution were added (Vector Laboratories) followed by detection of fluorescent signals using a confocal laser microscope (LSM 710; Carl Zeiss, Germany). Fluorescent intensity was measured by Zen software 2012 version (Carl Zeiss) and three random samples were taken per sample and measured using the Zen software.

### 2.4. Immunohistochemistry (3,3-Diaminobenzidine; DAB)

Blocks of paraffin-embedded aorta tissue were sectioned to a thickness of 7 µm, placed on a coating slide and dried at 40 °C for 24 h. Slides were deparaffinized and incubated in 0.3% H_2_O_2_ (Sigma-Aldrich) for 30 min. Then, slides were rinsed three times with PBS and incubated in normal animal serum to block non-specific binding, incubated with anti-pAMPK (abcam; dilution rate 1:200) or anti-PI3K antibodies (abcam, dilution rate 1:200) at 4 °C, followed by three additional rinses with PBS. Slides were then treated with biotinylated secondary antibodies from the ABC kit (Vector Laboratories, dilution rate 1:200), incubated for 1 h with blocking solution, and rinsed three times with PBS. Slides were left to react with 3,3′-diaminobenzidine (DAB) substrate for 15 min followed by mounting with a cover slip and DPX mounting solution (Sigma-Aldrich). Images were detected using a light microscope (Olympus, Japan) and quantification of the intensity of the brown color using Image J software (NIH, USA).

### 2.5. Quantitative Real Time Polymerase Chain Reaction (qRT-PCR)

qRT-PCR was used to quantify mRNA levels. Appropriate primers (listed in Table S1), distilled water and the RNA sample were mixed and placed in a 384-well plate followed by the addition of additional template complementary DNA (cDNA) and SYBR green (TAKARA, Japan). Mixed samples were validated using a PCR machine (Bio-Rad, CA, USA)

### 2.6. Enzyme-Linked Immunosorbent Assay (ELISA)

To measure serum level of adiponectin, an aliquot of the withdrawn blood (1 mL) was centrifuged and incubated in serum separator tubes (Becton Dickinson, USA) for 30 min. Samples were then centrifuged at 2000× *g* for 10 min and the supernatant moved into a new tube. The transparent serum specimens obtained were stored in a freezer at −80 °C. Adiponectin levels in serum and PVAT of each group were detected using the appropriate kit (abcam) according to the manufacturer’s instructions.

### 2.7. Histological Hematoxylin and Eosin (H & E) Staining

Blocks of paraffin-embedded aorta tissue were sectioned to a thickness of 7 µm, placed on a coating slide and dried at 40 °C for 24 h. Slides were deparaffinized and incubated in hematoxylin (DAKO, UK) for 1 min, eosin (Sigma-Aldrich) for 20 s followed by three rinses with PBS. Finally, slides were mounted with a cover slip and DPX mounting solution (Sigma-Aldrich) followed by detection with a light microscope (Olympus). The adipocyte size and ratio of white and brown adipocytes in PVAT were measured by using image J software (NIH, USA). Morphometrical analyses was conducted in blind and three operators conducted at least three replicates.

### 2.8. Statistical Analysis

The Kruskal–Wallis and Mann–Whitney U post-hoc tests were used to determine the significance of differences among the control, DIO/saline, DIO/ECE and DIO/PPB groups. Results are presented as mean ± SD, and statistical significance was accepted for *p*-values <0.05. The analysis was performed using SPSS version 22 (IBM Corporation, NY, USA). * (asterisk) indicates difference between some group vs. the control and $ (dollar) indicates difference between some group vs. DIO/saline. # (sharp) indicates difference between some group vs. DIO/ECE.

## 3. Results

### 3.1. ECE and PPB Increased the Proportion of BAT and Reduced Adipocyte Size

**Figure 1:**
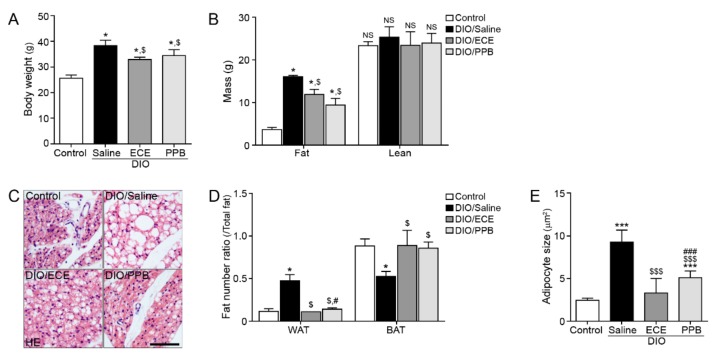
Regulatory effects of PPB and ECE on body weight, fat mass, perivascular fat abundance and adipocyte size in a diet-induced obesity mouse model. (**A**,**B**) Body weight and fat mass were measured before sacrifice. (**C**) Perivascular fat stained with H&E and (**D**) ratio of WAT and BAT in perivascular fat and (**E**) perivascular adipocyte size were measured using representative H&E stained images. Scale bar = 100 um; *, *p* < 0.05 and ***, *p* < 0.001 vs. the control group; $, *p* < 0.05 and $$$, *p* < 0.001 vs. DIO/Saline; #, *p* < 0.05 and ###, *p* < 0.001 vs. DIO/ECE. BAT, brown adipose tissue; ECE, *E. cava* extract; H&E, hematoxylin and eosin; PPB, pyrogallol-phloroglucinol-6,6-bieckol; WAT, white adipose tissue.

The body weight and fat mass of body composition were significantly higher in the DIO/saline group compared with the control group; however, body weight was lower in the DIO/ECE and DIO/PPB groups compared with the DIO/saline group (Figure 1A,B). In our study, the ratio of brown adipocytes to total adipocytes was significantly lower in the PVAT of the DIO/saline group compared with the control group, however this ratio was higher in the DIO/ECE and DIO/PPB groups compared with the DIO/saline group (Figure 1C,D). The ratio of white adipocytes to total adipocytes was significantly higher in the PVAT of the DIO/saline group compared with the control group, however this ratio was significantly lower in the DIO/ECE and DIO/PPB groups compared with the DIO/saline group (Figure 1C,D). Adipocyte size in PVAT was significantly larger in the DIO/saline group compared with the control group; however, the size of adipocytes was significantly smaller in the DIO/ECE and DIO/PPB groups compared with the DIO/saline group (Control < DIO/ECE < DIO/PPB < DIO/Saline) (Figure 1C,E). These results suggest that ECE and PPB decreased the proportion of brown adipocytes to total adipocytes of PVAT in DIO mice. In addition, ECE and PPB attenuated the increase in adipocyte size induced in DIO animals.

### 3.2. ECE and PPB Both Attenated M1/M2 Macrophage Polarization in PVAT of DIO Model

**Figure 2:**
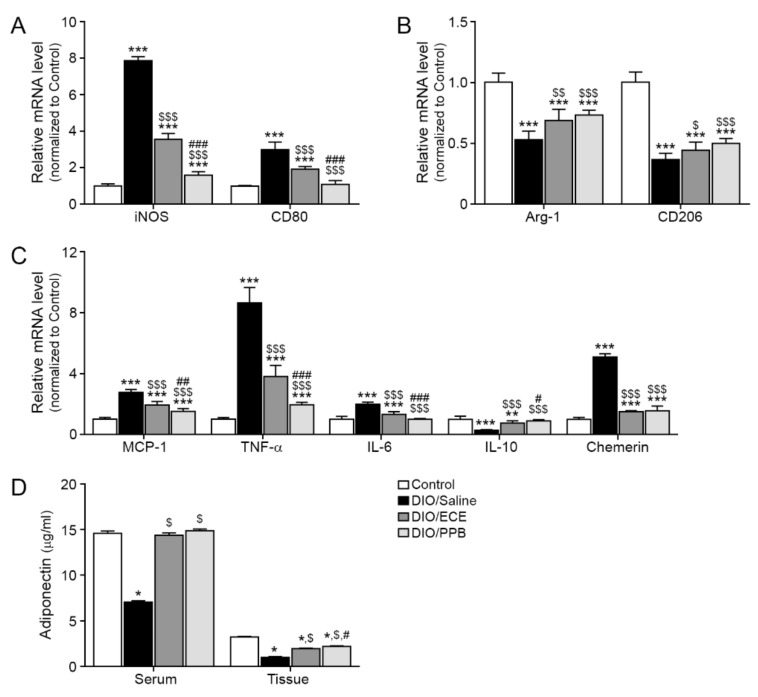
Regulatory effects of PPB and ECE on the abundance of M1/M2 macrophage markers, inflammatory factors and adiponectin in PVAT. (**A**) Expression of M1-type macrophage related genes including iNOS and CD80, and (**B**) M2-type macrophage related genes including Arg-1 and CD206 in PVAT of Control, DIO, DIO/ECE and DIO/PPB groups were validated by qRT-PCR. (**C**) Expression of inflammatory factors including MCP-1, TNF-α, IL-6, IL-10, and chemerin in PVAT of Control, DIO, DIO/ECE, and DIO/PPB groups were validated by qRT-PCR. (**D**) Adiponectin levels in blood serum and PVAT were measured by ELISA. All gene levels are normalized to those in control. *, *p* < 0.05, **, *p* < 0.01 and ***, *p* < 0.001 vs. the control group; $, *p* < 0.05; $$, *p* < 0.01; and $$$, *p* < 0.001 vs. DIO/Saline; #, *p* < 0.05; ##, *p* < 0.01; and ###, *p* < 0.001 vs. DIO/ECE. Arg-1, Arginase-1; ECE, *E. cava* extract; iNOS, Inducible nitric oxide synthase; IL-6, Interleukin-6; IL-1ß, Interleukin-1 beta; IL-10, Interleukin-10; MCP-1, Monocyte chemoattractant protein-1; PPB, pyrogallol-phloroglucinol-6,6-bieckol; PVAT, perivascular fat tissue; TNF-α; tumor necrosis factor-alpha.

Macrophages are typically classified into one of two phenotypes, M1 and M2; M1 macrophages are primarily located in active sites of inflammation positively contribute to inflammatory disease processes [48], while M2 macrophages are involved in anti-inflammatory pathways, tissue remodeling, and wound healing [48]. We evaluated the expression of inducible nitric oxide synthase (iNOS) and CD80, well-known markers of M1 macrophages and proinflammation [49], in PVAT. Significantly more iNOS and CD80 was observed in PVAT of the DIO/saline group compared with the control group, however their abundance was significantly lower in the DIO/ECE and DIO/PPB groups compared with the DIO/saline group (Control< DIO/PPB < DIO/ECE < DIO/Saline) (Figure 2A). The abundance of arginase-1 (Arg-1) and CD206, markers of M2 macrophages, was significantly lower in PVAT of the DIO/saline group compared with the control group, however their abundance was higher in the DIO/ECE and DIO/PPB groups compared with the DIO/saline group (Control > DIO/PPB > DIO/ECE > DIO/Saline) (Figure 2B).

### 3.3. ECE and PPB Attenated Inflammation in PVAT by DIO

The infiltration of macrophages into fat tissue is triggered by chemokines (e.g., MCP-1); HFD enhances MCP-1 expression in PVAT leading to aggravation of inflammation [19,26]. The abundance of MCP-1 was significantly greater in PVAT of the DIO/saline group compared with the control group, however this abundance was significantly less in the DIO/ECE and DIO/PPB groups compared with the DIO/saline group (Control < DIO/PPB < DIO/ECE < DIO/Saline) (Figure 2C).

In our study, the abundance of TNF-α and IL-6 were significantly higher in PVAT of the DIO/saline group compared with the control group, however their abundance was significantly lower in the DIO/ECE and DIO/PPB groups compared with the DIO/saline group (Control < DIO/PPB < DIO/ECE < DIO/Saline) (Figure 2C). The abundance of IL-10 was significantly lower in PVAT of the DIO/saline group compared with the control group, however there was more IL-10 in the DIO/ECE and DIO/PPB groups compared with the DIO/saline group (Figure 2C).

In our study, the abundance of chemerin was significantly higher in PVAT of the DIO/saline group compared with the control group, however its abundance was significantly lower in the DIO/ECE and DIO/PPB groups compared with the DIO/saline group (Control < DIO/PPB = DIO/ECE < DIO/Saline) (Figure 2C).

In our study, the adiponectin level in both PVAT and serum of mice in the DIO/saline group were higher compared with the control group, and those of the DIO/PPB and DIO/ECE groups were lower compared with mice in the DIO/saline group (Figure 2D). These results suggest that PPB and ECE can restore DIO-induced decreases in adiponectin.

### 3.4. ECE and PPB Attenuated ER Stress and Modulated the Browning Effect in the PVAT

**Figure 3:**
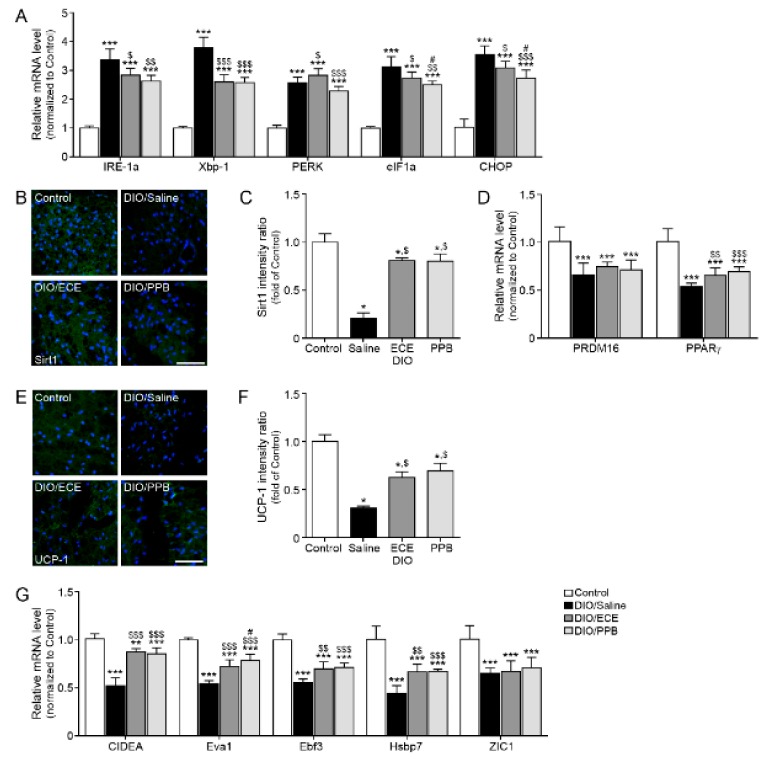
Modulating effects of PPB and ECE on endoplasmic reticulum (ER) stress and browning effect in PVAT (**A**) ER stress-related genes, including IRE-1α, Xbp-1, PERK, eIF1α, and CHOP in PVAT of Control, DIO, DIO/ECE, and DIO/PPB groups were validated by qRT-PCR. (**B**) Immunofluorescent images highlighting Sirt1 expression (green) and nuclei (blue; DAPI) in PVAT and (**C**) quantitative analysis graph also showing fluorescent intensity of Sirt1. (**D**) Sirt1-expression related genes including PRDM16 and PPARγ in PVAT of Control, DIO, DIO/ECE, and DIO/PPB groups were validated by qRT-PCR. (**E**) Immunofluorescent images indicating UCP-1 expression (green) and nuclei (blue; DAPI) in PVAT and (**F**) quantitative analysis graph also revealing fluorescent intensity of UCP-1. (**G**) Brown fat markers, including CIDEA, Eva1, Ebf3, HsBP7, ZIC1 in PVAT of Control, DIO, DIO/ECE, and DIO/PPB groups were validated by qRT-PCR. All gene levels are normalized to those in control. Scale bar = 200 μm, *, *p* < 0.05; **, *p* < 0.01; and ***, *p* < 0.001 vs. the control group; $, *p* < 0.05; $$, *p* < 0.01; and $$$, *p* < 0.001 vs. DIO/Saline; #, *p* < 0.05; ##, *p* < 0.01; and ###, *p* < 0.001 vs. DIO/ECE. CIDEA, cell death-inducing DNA fragmentation factor alpha-like effector A; CHOP, C/EBP Homologous Protein; DIO, diet-induced obesity; Ebf3, EBF Transcription Factor 3; ECE, *E. cava* extract; eIF1α, eukaryotic translation initiation factor 1; IRE-1α, inositol-requiring transmembrane kinase/endoribonuclease 1α; PERK, protein kinase RNA-like endoplasmic reticulum kinase; PPB, pyrogallol-phloroglucinol-6,6-bieckol; PVAT, perivascular fat tissue; UCP-1, uncoupling protein 1; Xbp-1, X-box binding protein 1; Zinc1; Zinc finger of the cerebellum 1.

In our study, the ER stress markers inositol-requiring enzyme 1 α (IRE-1α), X-box-binding protein-1 (Xbp-1), PERK, eukaryotic translation initiation factor 1a (eIF1a), and C/EBP-homologous protein (CHOP) were increased in the DIO/saline group compared with the control group; however, their abundance was lower in the DIO/PPB and DIO/ECE groups compared with the DIO/saline group (Figure 3A). The abundance of Sirt1 (green signal), PR-domain containing 16 (Prdm16), and PPARγ were lower in the DIO/saline group compared with the control group, however their levels in the DIO/PPB and DIO/ECE groups were higher compared with the DIO/saline group (Figure 3B–D). The abundance of UCP-1 (green signal) in the DIO/saline group was lower in the DIO/saline group compared with the control group, however the UCP-1 level in DIO/PPB and DIO/ECE groups were higher compared with the DIO/saline group (Figure 3E,F).

The abundance of BAT markers (i.e., cell death-inducing DNA fragmentation factor alpha-like effector A; CIDEA, epithelial like antigen-1; Eva1, EBF transcription factor 3; Ebf3, heat shock protein family B member 7; Hsbp7, Zinc finger of the cerebellum 1; ZIC1), were lowest in the DIO/saline group (Figure 3G), however, their abundance of these markers in the DIO/PPB and DIO/ECE groups was higher compared with the DIO/saline group. Thus, our results revealed that ECE and PPB decreased HFD-mediated ER stress and attenuated ER-mediated decreases in the expression of Sirt1 and PPARγ. The ECE and PPB-induced protective effects (i.e., reductions in the abundance of Sirt1 and PPARγ) led to a restoration of UCP-1 expression and browning processes in PVAT; these protective effects were more potent in the PPB group compared with the ECE group.

### 3.5. ECE and PPB Attenuate Endothelial Cell Dysfunction

**Figure 4:**
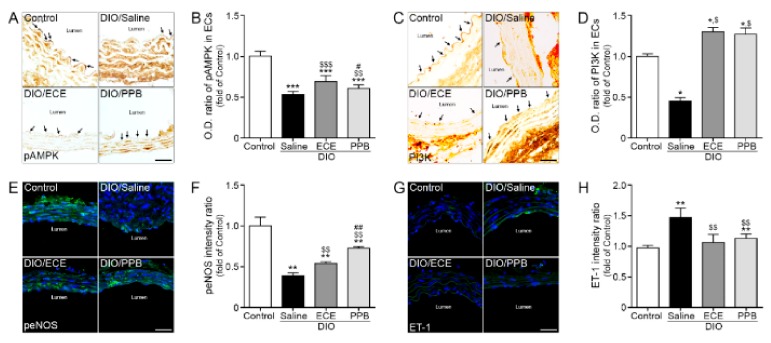
Modulating effects of PPB and ECE on endothelial cell dysfunction (**A**,**C**) Immunohistochemical images showing pAMPK and PI3K expression (brown, arrow) in the endothelial cell layer and (**B**,**D**) quantitative graph showing optical density (O.D.) of pAMPK and PI3K. (**E**) Immunofluorescent images showing peNOS expression (green) and nuclei (blue; DAPI) in the endothelial cell layer and (**F**) quantitative analysis graph also showing fluorescent intensity of peNOS. (**G**) Immunofluorescent images showing ET-1 expression (green) and nuclei (blue; DAPI) in the endothelial cell layer and (**H**) quantitative analysis graph also showing fluorescent intensity of ET-1. Scale bar = 150 μm, *, *p* < 0.05, **, *p* < 0.01 and ***,*p* < 0.001 vs. the control group; $, *p* < 0.05; $$, *p* < 0.01; and $$$, *p* < 0.001 vs. DIO/Saline; #,*p* < 0.05; ##,*p* < 0.01; vs. DIO/ECE, DIO, diet-induced obesity; ECE, *E. cava* extract; ET-1, endothelin-1; pAMPK, phosphorylated AMP-activated protein kinase; peNOS, phosphorylated endothelial NOS; PI3K, phosphoinositide 3-kinases; PPB, pyrogallol-phloroglucinol-6,6-bieckol.

In our study, the abundance of pAMPK, PI3K, and epNOS were lower in the DIO/saline group compared with the control group, however the abundance of pAMPK, PI3K (brown color, arrow) was higher in the DIO/PPB and DIO/ECE groups compared with the DIO/saline group (Figure 4A–D). As well as pAMPK-PI3K expression, expression of peNOS and ET-1 of EC (green signal) detected and these results suggest that HFD-induced EC dysfunction which manifest as downregulation of the pAMPK-PI3K-peNOS pathway is attenuated by PPB or ECE in PVAT (Figure 4A–F). In our study, the abundance of ET-1 was higher in the DIO/saline group compared with the control group, however it was lower in the DIO/PPB and DIO/ECE groups compared with the DIO/saline group (Figure 4G,H). These results suggest that PPB and ECE decrease the abundance of HFD-induced ET-1 in PVAT.

## 4. Discussion

Obesity is well known to increase the size and abundance of WAT in PVAT [19,22,23,24] which limits oxygen perfusion in PVAT and leads to hypoxia [21]. Ultimately, the changes referenced here initiate inflammation in the adipose tissue [21]. As well as adipocyte changes, macrophage polarization also important in PVAT of HFD mouse. In our previous study, we showed that a HFD increased the number of M1 macrophages and decreased the number of M2 macrophages in PVAT [50]. Monocytes are polarized into M1 or M2 phenotypes according to the microenvironmental conditions [48]. M1 polarization is induced by lipopolysaccharide and interferon-γ (IFN-γ) or other pro-inflammatory cytokines, while M2 is generated by IL-4, IL-13, or IL-10 [48]. Obesity leads to the polarization of macrophages from the M2 to the M1 phenotype in fat tissues and aggravate inflammation in the fat tissue by increasing the proportion of M1 macrophages which secrete pro-inflammatory cytokines such as TNF-α and IL-1β [48]. Our present study revealed that ECE and PPB attenuate the DIO-induced increase and decrease in M1 and M2 macrophages, respectively; PPB better attenuated these changes compared with ECE and the oral administration concentration of ECE (70 mg/kg/day) and PPB (2.5 mg/kg/day) used in this study is safe concentrations in which no abnormal behavior and toxicity of animals were identified in previous study [45].

Infiltration of macrophages into PVAT leads to increasing production or secretion of adipokines and cytokines and a subsequent increase in inflammation. HFD leads to increases in the abundance of pro-inflammatory cytokines such as IL-6 and TNF-α in PVAT and decreases the abundance of anti-inflammatory adipokines or cytokines such as adiponectin and IL-10 [19,26,51]. These changes to the cytokine profile promote a dysregulation of biomolecule production in PVAT leading to vascular dysfunction [8]. TNF-α and IL-6 act not only as aggravation factors of inflammation but also as vasoconstrictors by increasing endothelin signaling or reducing NO production and endothelium-dependent relaxation which are known to be more prominent in obese patients [13,14,15].

The abundance of IL-6 in human coronary PVAT increases as a result of a HFD [51]. IL-6 secreted from PVAT binds to its cognate receptor and leads to EC dysfunction by reducing bioavailable NO [52]. PVAT could secrete chemerin and the primary chemerin receptor, chemerin receptor 23 (ChemR23), was expressed both in the SMC of tunica media and EC layer of the arteries [53]. The ChemR23 agonist chemerin-9 induced receptor-dependent or concentration-dependent contraction in isolated rat thoracic aorta, superior mesenteric artery, and mesenteric resistance artery [54]. Chemerin-induced vasocontraction was significantly increased when nitric oxide synthase (NOS) was inhibited, or ECs were mechanically removed which suggested EC dysfunction [53]. In addition, chemerin secreted from PAVT increase sympathetic contraction via its receptor, which is co-localized with tyrosine hydrolase in sympathetic nerves of rat superior mesenteric artery [53]. Those studies suggested that chemerin, a vasoactive PVAT factor, may serve as a connector between obesity and a change in arterial tone such as hypertension [53].

Adiponectin secreted from PVAT induced vasodilation through multiple mechanisms. First, adiponectin stimulates NO release from adjacent adipocytes. Then, adipocyte-derived NO activates large-conductance Ca^2+^-activated K^+^ channels opening in VSMC [54] and leads to vasodilation. Adiponectin also stimulates NO production from ECs by enhancing the binding of heat shock protein 90 (Hsp90) to endothelial NOS (eNOS) [55], by increasing eNOS phosphorylation at serine 1177 by either PI3K/Akt [55,56,57] or AMP-activated kinase (AMPK) [58], and by enhancing the biosynthesis of tetrahydrobiopterin, an essential cofactor of eNOS [57].

Many studies have confirmed the anticontractile effect of PVAT induced by adiponectin. In adiponectin-deficient mice, vasoconstriction is significantly reduced [59,60], and the anticontractile function of PVAT is largely abolished by inhibiting adiponectin receptors [60]. In patients with coronary artery disease, the abundance of adiponectin is positively correlated with EC function [57]; genome-wide association studies have also shown that lower levels of circulating adiponectin are associated with EC dysfunction [57]. The activation of UCP-1 and induction of genes encoding UCP-1 enhance uptake of lipids and glucose from the circulation to induce oxidation and thermogenesis [61,62]. Many studies have shown that beige adipocytes are an inducible brown-like white adipocytes and they are developed among WAT by β-adrenergic receptor stimulation [63,64]. The process which converts WAT into beige adipocytes is called browning [63,64]. During browning, the transcription of UCP-1 is tightly regulated, and thus is frequently considered a marker of adipose tissue browning [65,66]. Beige adipocytes may also undergo a unique plasticity process (i.e., reversal of browning) and change into WAT [67,68,69]. Peroxisome proliferator-activated receptor (PPARγ) known to be an important activator of UCP-1 expression [65,70] and PPARγ protein level is decreased under ER-stress conditions [71]. Thus, ER stress downregulates PPARγ and leads to a reduced expression of UCP-1, thus hindering WAT browning [71].

In addition, Sirtuin 1 (Sirt1), NAD+-dependent deacetylase and that is very important to metabolic control, is involved in WAT browning processes. The activation of Sirt1 in WAT leads to the deacetylation of PPARγ [72,73] which leads to the creation of a transcription complex with PRDM16 and peroxisome proliferator-activated receptor gamma coactivator 1-alpha (PGC-1α) [74,75]. Complexes of PPARγ with PRDM16 and PGC-1α promote the transcription of genes which induce the formation of beige adipocytes [74,75]. PPARγ also inhibits the transcription of genes involved in WAT formation [74,76]. Thus, Sirt1 promotes enhancement of BAT-gene transcription and suppression of WAT genes through PPARγ. Furthermore, Sirt1 decreases fat storage, increases lipolysis in adipose tissue and protects against obesity-induced inflammation [75,77]. In BAT of obese mice, the abundance of Sirt1 was reduced by increased ER stress, leading to BAT apoptosis [35] and phlorotannins from *E. cava* attenuates palmitate-induced endoplasmic reticulum stress [78]. Thus, ER stress contributes to: (i) the dysregulation of Sirt1 and PPARγ, (ii) reductions in the abundance of UCP-1, (iii) browning signal expression [79], and (iv) apoptosis in BAT.

AMPK is expressed in both EC and VSMC. In EC, AMPK activates the phosphorylation of eNOS which leads to the production of NO [77,80] through protein kinase B (Akt) [81] or PI3K-Akt pathways [82]. EC dysfunction is characterized by reduced eNOS availability leading to a reduced endothelium-dependent vasodilatory response [83]. Endothelin-1 (ET-1), a powerful vasoconstrictor, is mostly expressed in the EC [84,85]. Furthermore, ET-1 promotes migration and proliferation of VSMCs which are related with intimal hyperplasia [86]. The ET-1 is involved in the production of vascular reactive oxygen species and acts as a pro-inflammatory cytokine, inducing atherosclerosis [87,88]. ET-1 induces an increase in the abundance of adhesion molecules such as MCP-1, migration of macrophages, and activation of pro-inflammatory M1-type macrophages, processes important in the pathophysiology of atherosclerosis [89].

## 5. Conclusions

Modulating PVAT by increasing the proportion of thermogenic brown or beige adipocytes might be a feasible approach to attenuate local inflammation and reduce the risk of cardiovascular diseases [90]. The results presented here reveal that ECE and PPB attenuated inflammation and subsequent PVAT dysfunction. By reducing PVAT dysfunction, the abundance of vasoconstrictors was decreased and the abundance of vasodilators or anti-inflammatory cytokines/adipokines were increased. In addition, ECE and PPB attenuated HFD-induced decreases in brown adipocyte gene expression, thus protecting against EC dysfunction also induced by HFD (Figure S1).

## Supplementary Materials

The following are available online at https://www.mdpi.com/2072-6643/11/11/2795/s1, Table S1: List of primer for qRT-PCR; Figure S1: Summarized scheme images for experiments.

## Abbreviations

Arg-1	Arginase-1
CIDEA	Cell death-inducing DNA fragmentation factor alpha-like effector A
CHOP	C/EBP Homologous Protein
DIO	Diet-induced obesity
Ebf3	EBF Transcription Factor 3
ECE	*E. cava* extract
eIF1α	Eukaryotic translation initiation factor 1
ET-1	Endothelin-1
iNOS	Inducible nitric oxide synthase
IL-6	Interleukin-6
IL-1ß	Interleukin-1 beta
IL-10	Interleukin-10
IRE-1α	Inositol-requiring transmembrane kinase/endoribonuclease 1α
MCP-1	Monocyte chemoattractant protein-1
pAMPK	Phosphorylated AMP-activated protein kinase
peNOS	Phosphorylated Endothelial NOS
PERK	Protein kinase RNA-like endoplasmic reticulum kinase
PI3K	Phosphoinositide 3-kinases
PPB	Pyrogallol-phloroglucinol-6,6-bieckol
PVAT	Perivascular fat tissue
UCP-1	Uncoupling protein 1
Xbp-1	X-box binding protein 1
Zinc1	Zinc finger of the cerebellum 1
PPB	Pyrogallol-phloroglucinol-6,6-bieckol
TNF-α	Tumor necrosis factor-alpha

## References

[1] Kim S., Moustaid-Moussa N. (2000). Secretory, endocrine and autocrine/paracrine function of the adipocyte. J. Nutr..

[2] Smith R.E., Horwitz B.A. (1969). Brown fat and thermogenesis. Phsiol. Rev..

[3] Ozen G., Daci A., Norel X., Topal G. (2015). Human perivascular adipose tissue dysfunction as a cause of vascular disease: Focus on vascular tone and wall remodeling. Eur. J. Pharmacol..

[4] Victorio J.A., Fontes M.T., Rossoni L.V., Davel A.P. (2016). Different anti-contractile function and nitric oxide production of thoracic and abdominal perivascular adipose tissues. Front. Physiol..

[5] Hildebrand S., Stümer J., Pfeifer A. (2018). PVAT and its relation to brown, beige, and white adipose tissue in development and function. Front. Physiol..

[6] Gálvez-Prieto B., Bolbrinker J., Stucchi P., de Las Heras A.I., Merino B., Arribas S., Ruiz-Gayo M., Huber M., Wehland M., Kreutz R. (2008). Comparative expression analysis of the renin-angiotensin system components between white and brown perivascular adipose tissue. J. Endocrinol..

[7] Brown N.K., Zhou Z., Zhang J., Zeng R., Wu J., Eitzman D.T., Chen Y.E., Chang L. (2014). Perivascular adipose tissue in vascular function and disease: A review of current research and animal models. Arterioscler. Thromb. Vasc. Biol..

[8] Almabrouk T.A., Ewart M.A., Salt I.P., Kennedy S. (2014). Perivascular fat, AMP-activated protein kinase and vascular diseases. Br. J. Pharmacol..

[9] Villacorta L., Chang L. (2015). The role of perivascular adipose tissue in vasoconstriction, arterial stiffness, and aneurysm. Horm. Mol. Biol. Clin. Investig..

[10] Li Y., Mihara K., Saifeddine M., Krawetz A., Lau D.C., Li H., Ding H., Triggle C.R., Hollenberg M.D. (2011). Perivascular adipose tissue-derived relaxing factors: Release by peptide agonists via proteinase-activated receptor-2 (PAR2) and non-PAR2 mechanisms. Br. J. Pharmacol..

[11] Gollasch M. (2012). Vasodilator signals from perivascular adipose tissue. Br. J. Pharmacol..

[12] Gollasch M. (2017). Adipose-vascular coupling and potential therapeutics. Annu. Rev. Pharmacol. Toxicol..

[13] Greenberg S., Xie J., Wang Y., Cai B., Kolls J., Nelson S., Hyman A., Summer W.R., Lippton H. (1993). Tumor necrosis factor-alpha inhibits endothelium-dependent relaxation. J. Appl. Physiol..

[14] Orshal J.M., Khalil R.A. (2004). Interleukin-6 impairs endothelium-dependent NO-cGMP-mediated relaxation and enhances contraction in systemic vessels of pregnant rats. Am. J. Physiol. Regul. Integr. Comp. Physiol..

[15] Virdis A., Duranti E., Rossi C., Dell’Agnello U., Santini E., Anselmino M., Chiarugi M., Taddei S., Solini A. (2015). Tumour necrosis factor-alpha participates on the endothelin-1/nitric oxide imbalance in small arteries from obese patients: Role of perivascular adipose tissue. Eur. Heart J..

[16] Aghamohammadzadeh R., Withers S., Lynch F., Greenstein A., Malik R., Heagerty A. (2012). Perivascular adipose tissue from human systemic and coronary vessels: The emergence of a new pharmacotherapeutic target. Br. J. Pharmacol..

[17] Fitzgibbons T.P., Czech M.P. (2014). Epicardial and perivascular adipose tissues and their influence on cardiovascular disease: Basic mechanisms and clinical associations. J. Am. Heart Assoc..

[18] Nosalski R., Guzik T.J. (2017). Perivascular adipose tissue inflammation in vascular disease. Br. J. Pharmacol..

[19] Ketonen J., Shi J., Martonen E., Mervaala E. (2010). Periadventitial adipose tissue promotes endothelial dysfunction via oxidative stress in diet-induced obese C57Bl/6 mice. Circ. J..

[20] Ma L., Ma S., He H., Yang D., Chen X., Luo Z., Liu D., Zhu Z. (2010). Perivascular fat-mediated vascular dysfunction and remodeling through the AMPK/mTOR pathway in high-fat diet-induced obese rats. Hypertens. Res..

[21] Achike F.I., To N.H., Wang H., Kwan C.Y. (2011). Obesity, metabolic syndrome, adipocytes and vascular function: A holistic viewpoint. Clin. Exp. Pharmacol. Physiol..

[22] Hosogai N., Fukuhara A., Oshima K., Miyata Y., Tanaka S., Segawa K., Furukawa S., Tochino Y., Komuro R., Matsuda M. (2007). Adipose tissue hypoxia in obesity and its impact on adipocytokine dysregulation. Diabetes.

[23] Greenstein A.S., Khavandi K., Withers S.B., Sonoyama K., Clancy O., Jeziorska M., Laing I., Yates A.P., Pemberton P.W., Malik R.A. (2009). Local inflammation and hypoxia abolish the protective anticontractile properties of perivascular fat in obese patients. Circulation.

[24] Gustafson B., Hammarstedt A., Andersson C.X., Smith U. (2007). Inflamed adipose tissue: A culprit underlying the metabolic syndrome and atherosclerosis. Arterioscler. Thromb. Vasc. Biol..

[25] Chatterjee T.K., Aronow B.J., Tong W.S., Manka D., Tang Y., Bogdanov V.Y., Unruh D., Blomkalns A.L., Piegore M.G., Weintraub D.S. (2013). Human coronary artery perivascular adipocytes overexpress genes responsible for regulating vascular morphology, inflammation, and hemostasis. Physiol. Genom..

[26] Xia N., Horke S., Habermeier A., Closs E.I., Reifenberg G., Gericke A., Mikhed Y., Münzel T., Daiber A., Förstermann U. (2016). Uncoupling of endothelial nitric oxide synthase in perivascular adipose tissue of diet-induced obese mice. Arterioscler. Thromb. Vasc. Biol..

[27] Gil-Ortega M., Condezo-Hoyos L., García-Prieto C.F., Arribas S.M., González M.C., Aranguez I., Ruiz-Gayo M., Somoza B., Fernández-Alfonso M.S. (2014). Imbalance between pro and anti-oxidant mechanisms in perivascular adipose tissue aggravates long-term high-fat diet-derived endothelial dysfunction. PLoS ONE.

[28] Aghamohammadzadeh R., Unwin R.D., Greenstein A.S., Heagerty A.M. (2015). Effects of obesity on perivascular adipose tissue vasorelaxant function: Nitric oxide, inflammation and elevated systemic blood pressure. J. Vasc. Res..

[29] Schäfer K., Drosos I., Konstantinides S. (2017). Perivascular adipose tissue: Epiphenomenon or local risk factor?. Int. J. Obes..

[30] Fitzgibbons T.P., Kogan S., Aouadi M., Hendricks G.M., Straubhaar J., Czech M.P. (2011). Similarity of mouse perivascular and brown adipose tissues and their resistance to diet-induced inflammation. Am. J. Physiol. Heart Circ. Physiol..

[31] Chang L., Villacorta L., Li R., Hamblin M., Xu W., Dou C., Zhang J., Wu J., Zeng R., Chen Y.E. (2012). Loss of perivascular adipose tissue on peroxisome proliferator-activated receptor-γ deletion in smooth muscle cells impairs intravascular thermoregulation and enhances atherosclerosis. Circulation.

[32] Zhang K., Kaufman R.J. (2008). From endoplasmic-reticulum stress to the inflammatory response. Nature.

[33] Yin J., Wang Y., Gu L., Fan N., Ma Y., Peng Y. (2015). Palmitate induces endoplasmic reticulum stress and autophagy in mature adipocytes: Implications for apoptosis and inflammation. Int. J. Mol. Med..

[34] Xu X., Chen Y., Song J., Hou F., Ma X., Liu B., Huang F. (2018). Mangiferin suppresses endoplasmic reticulum stress in perivascular adipose tissue and prevents insulin resistance in the endothelium. Eur. J. Nutr..

[35] Liu Z., Gu H., Gan L., Xu Y., Feng F., Saeed M., Sun C. (2017). Reducing Smad3/ATF4 was essential for Sirt1 inhibiting ER stress-induced apoptosis in mice brown adipose tissue. Oncotarget.

[36] Picard F., Kurtev M., Chung N., Topark-Ngarm A., Senawong T., Machado De Oliveira R., Leid M., McBurney M.W., Guarente L. (2004). Sirt1 promotes fat mobilization in white adipocytes by repressing PPAR-gamma. Nature.

[37] Xu C., Bai B., Fan P., Cai Y., Huang B., Law I.K., Liu L., Xu A., Tung C., Li X. (2013). Selective overexpression of human SIRT1 in adipose tissue enhances energy homeostasis and prevents the deterioration of insulin sensitivity with ageing in mice. Am. J. Transl. Res..

[38] Xu F., Lin B., Zheng X., Chen Z., Cao H., Xu H., Liang H., Weng J. (2016). GLP-1 receptor agonist promotes brown remodelling in mouse white adipose tissue through SIRT1. Diabetologia.

[39] Wang L., Teng R., Di L., Rogers H., Wu H., Kopp J.B., Noguchi C.T. (2013). PPARα and Sirt1 mediate erythropoietin action in increasing metabolic activity and browning of white adipocytes to protect against obesity and metabolic disorders. Diabetes.

[40] Fu T., Seok S., Choi S., Huang Z., Suino-Powell K., Xu H.E., Kemper B., Kemper J.K. (2014). MicroRNA 34a inhibits beige and brown fat formation in obesity in part by suppressing adipocyte fibroblast growth factor 21 signaling and SIRT1 function. Mol. Cell. Biol..

[41] Yang Y.I., Woo J.H., Seo Y.J., Lee K.T., Lim Y., Choi J.H. (2016). Protective effect of brown alga phlorotannins against hyper-inflammatory responses in lipopolysaccharide-induced sepsis models. J. Agric. Food Chem..

[42] Yang Y.I., Shin H.C., Kim S.H., Park W.Y., Lee K.T., Choi J.H. (2012). 6,6’-Bieckol, isolated from marine alga Ecklonia cava, suppressed LPS-induced nitric oxide and PGE_2_ production and inflammatory cytokine expression in macrophages: The inhibition of NFκB. Int. Immunopharmacol..

[43] Lee M.S., Shin T., Utsuki T., Choi J.S., Byun D.S., Kim H.R. (2012). Isolation and identification of phlorotannins from Ecklonia stolonifera with antioxidant and hepatoprotective properties in tacrine-treated HepG2 cells. J. Agric. Food Chem..

[44] Choi H.S., Jeon H.J., Lee O.H., Lee B.Y. (2015). Dieckol, a major phlorotannin in Ecklonia cava, suppresses lipid accumulation in the adipocytes of high-fat diet-fed zebrafish and mice: Inhibition of early adipogenesis via cell-cycle arrest and AMPKα activation. Mol. Nutr. Food Res..

[45] Son M., Oh S., Lee H.S., Ryu B., Jiang Y., Jang J.T., Jeon Y.J., Byun K. (2019). Pyrogallol-phloroglucinol-6,6′-bieckol from Ecklonia cava improved blood circulation in diet-induced obese and diet-induced hypertension mouse models. Mar. Drugs.

[46] Oh S., Son M., Lee H.S., Kim H.S., Jeon Y.J., Byun K. (2018). Protective effect of pyrogallol-phloroglucinol-6,6-bieckol from Ecklonia cava on monocyte-associated vascular dysfunction. Mar. Drugs.

[47] Lee J.H., Ko J.Y., Oh J.Y., Kim C.Y., Lee H.J., Kim J., Jeon Y.J. (2014). Preparative isolation and purification of phlorotannins from Ecklonia cava using centrifugal partition chromatography by one-step. Food Chem..

[48] Lee B.C., Lee J. (2014). Cellular and molecular players in adipose tissue inflammation in the development of obesity-induced insulin resistance. Biochim. Biophys. Acta.

[49] Kang H., Zhang K., Wong D.S.H., Han F., Li B., Bian L. (2018). Near-infrared light-controlled regulation of intracellular calcium to modulate macrophage polarization. Biomaterials.

[50] Oh S., Ahn H., Park H., Lee J.I., Park K.Y., Hwang D., Lee S., Son K.H., Byun K. (2019). The attenuating effects of pyridoxamine on adipocyte hypertrophy and inflammation differ by adipocyte location. J. Nutr. Biochem..

[51] Chatterjee T.K., Stoll L.L., Denning G.M., Harrelson A., Blomkalns A.L., Idelman G., Rothenberg F.G., Neltner B., Romig-Martin S.A., Dickson E.W. (2009). Proinflammatory phenotype of perivascular adipocytes: Influence of high-fat feeding. Circ. Res..

[52] Qu D., Liu J., Lau C.W., Huang Y. (2014). IL-6 in diabetes and cardiovascular complications. Br. J. Pharmacol..

[53] Watts S.W., Dorrance A.M., Penfold M.E., Rourke J.L., Sinal C.J., Seitz B., Sullivan T.J., Charvat T.T., Thompson J.M., Burnett R. (2013). Chemerin connects fat to arterial contraction. Arterioscler. Thromb. Vasc. Biol..

[54] Darios E.S., Winner B.M., Charvat T., Krasinksi A., Punna S., Watts S.W. (2016). The adipokine chemerin amplifies electrical field-stimulated contraction in the isolated rat superior mesenteric artery. Am. J. Physiol. Heart Circ. Physiol..

[55] Xi W., Satoh H., Kase H., Suzuki K., Hattori Y. (2005). Stimulated HSP90 binding to eNOS and activation of the PI3-Akt pathway contribute to globular adiponectin-induced NO production: Vasorelaxation in response to globular adiponectin. Biochem. Biophys. Res. Commun..

[56] Cerqueira F.M., Brandizzi L.I., Cunha F.M., Laurindo F.R., Kowaltowski A.J. (2012). Serum from calorie-restricted rats activates vascular cell eNOS through enhanced insulin signaling mediated by adiponectin. PLoS ONE.

[57] Margaritis M., Antonopoulos A.S., Digby J., Lee R., Reilly S., Coutinho P., Shirodaria C., Sayeed R., Petrou M., de Silva R. (2013). Interactions between vascular wall and perivascular adipose tissue reveal novel roles for adiponectin in the regulation of endothelial nitric oxide synthase function in human vessels. Circulation.

[58] Withers S.B., Bussey C.E., Saxton S.N., Melrose H.M., Watkins A.E., Heagerty A.M. (2014). Mechanisms of adiponectin-associated perivascular function in vascular disease. Arterioscler. Thromb. Vasc. Biol..

[59] Lynch F.M., Withers S.B., Yao Z., Werner M.E., Edwards G., Weston A.H., Heagerty A.M. (2013). Perivascular adipose tissue-derived adiponectin activates BK(Ca) channels to induce anticontractile responses. Am. J. Physiol. Heart Circ. Physiol..

[60] Withers S.B., Simpson L., Fattah S., Werner M.E., Heagerty A.M. (2014). cGMP-dependent protein kinase (PKG) mediates the anticontractile capacity of perivascular adipose tissue. Cardiovasc. Res..

[61] Giralt M., Villarroya F. (2013). White, brown, beige/brite: Different adipose cells for different functions?. Endocrinology.

[62] Marlatt K.L., Ravussin E. (2017). Brown adipose tissue: An update on recent findings. Curr. Obes. Rep..

[63] Harms M., Seale P. (2013). Brown and beige fat: Development, function and therapeutic potential. Nat. Med..

[64] Vitali A., Murano I., Zingaretti M.C., Frontini A., Ricquier D., Cinti S. (2012). The adipose organ of obesity-prone C57BL/6J mice is composed of mixed white and brown adipocytes. J. Lipid Res..

[65] Villarroya F., Iglesias R., Giralt M. (2007). PPARs in the control of uncoupling proteins gene expression. PPAR Res..

[66] Kalinovich A.V., de Jong J.M., Cannon B., Nedergaard J. (2017). UCP1 in adipose tissues: Two steps to full browning. Biochimie.

[67] Okla M., Wang W., Kang I., Pashaj A., Carr T., Chung S. (2015). Activation of Toll-like receptor 4 (TLR4) attenuates adaptive thermogenesis via endoplasmic reticulum stress. J. Biol. Chem..

[68] Lee Y.K., Cowan C.A. (2013). White to brite adipocyte transition and back again. Nat. Cell Biol..

[69] Rosenwald M., Perdikari A., Rülicke T., Wolfrum C. (2013). Bi-directional interconversion of brite and white adipocytes. Nat. Cell Biol..

[70] Xue B., Coulter A., Rim J.S., Koza R.A., Kozak L.P. (2005). Transcriptional synergy and the regulation of Ucp1 during brown adipocyte induction in white fat depots. Mol. Cell. Biol..

[71] Yuliana A., Daijo A., Jheng H.F., Kwon J., Nomura W., Takahashi H., Ara T., Kawada T., Goto T. (2019). Endoplasmic reticulum stress impaired uncoupling protein 1 expression via the suppression of peroxisome proliferator-activated receptor γ binding activity in mice beige adipocytes. Int. J. Mol. Sci..

[72] Qiang L., Wang L., Kon N., Zhao W., Lee S., Zhang Y., Rosenbaum M., Zhao Y., Gu W., Farmer S.R. (2012). Brown remodeling of white adipose tissue by SirT1-dependent deacetylation of Pparγ. Cell.

[73] Han L., Zhou R., Niu J., McNutt M.A., Wang P., Tong T. (2010). SIRT1 is regulated by a PPAR{γ}-SIRT1 negative feedback loop associated with senescence. Nucleic Acids Res..

[74] Lo K.A., Sun L. (2013). Turning WAT into BAT: A review on regulators controlling the browning of white adipocytes. Biosci. Rep..

[75] Seale P., Bjork B., Yang W., Kajimura S., Chin S., Kuang S., Scimè A., Devarakonda S., Conroe H.M., Erdjument-Bromage H. (2008). PRDM16 controls a brown fat/skeletal muscle switch. Nature.

[76] Vernochet C., Peres S.B., Davis K.E., McDonald M.E., Qiang L., Wang H., Scherer P.E., Farmer S.R. (2009). C/EBPalpha and the corepressors CtBP1 and CtBP2 regulate repression of select visceral white adipose genes during induction of the brown phenotype in white adipocytes by peroxisome proliferator-activated receptor gamma agonists. Mol. Cell. Biol..

[77] Lone J., Parray H.A., Yun J.W. (2018). Nobiletin induces brown adipocyte-like phenotype and ameliorates stress in 3T3-L1 adipocytes. Biochimie.

[78] Oh S., Son M., Choi J., Choi C.H., Park K.Y., Son K.H., Byun K. (2019). Phlorotannins from ecklonia cava attenuates palmitate-induced endoplasmic reticulum stress and leptin resistance in hypothalamic neurons. Mar. Drugs.

[79] Nascimento E.B., Boon M.R., van Marken Lichtenbelt W.D. (2014). Fat cells gain new identities. Sci. Transl. Med..

[80] Chen Z., Peng I.C., Sun W., Su M.I., Hsu P.H., Fu Y., Zhu Y., DeFea K., Pan S., Tsai M.D. (2009). AMP-activated protein kinase functionally phosphorylates endothelial nitric oxide synthase Ser633. Circ. Res..

[81] Levine Y.C., Li G.K., Michel T. (2007). Agonist-modulated regulation of AMP-activated protein kinase (AMPK) in endothelial cells. Evidence for an AMPK -> Rac1 -> Akt -> endothelial nitric-oxide synthase pathway. J. Biol. Chem..

[82] Ning W.H., Zhao K. (2013). Propionyl-L-carnitine induces eNOS activation and nitric oxide synthesis in endothelial cells via PI3 and Akt kinases. Vascul. Pharmacol..

[83] Li X., Li J., Li Z., Sang Y., Niu Y., Zhang Q., Ding H., Yin S. (2016). Fucoidan from Undaria pinnatifida prevents vascular dysfunction through PI3K/Akt/eNOS-dependent mechanisms in the l-NAME-induced hypertensive rat model. Food Funct..

[84] Yanagisawa M., Kurihara H., Kimura S., Tomobe Y., Kobayashi M., Mitsui Y., Yazaki Y., Goto K., Masaki T. (1988). A novel potent vasoconstrictor peptide produced by vascular endothelial cells. Nature.

[85] Böhm F., Pernow J. (2007). The importance of endothelin-1 for vascular dysfunction in cardiovascular disease. Cardiovasc. Res..

[86] Ivey M.E., Osman N., Little P.J. (2008). Endothelin-1 signalling in vascular smooth muscle: Pathways controlling cellular functions associated with atherosclerosis. Atherosclerosis.

[87] Browatzki M., Schmidt J., Kübler W., Kranzhöfer R. (2000). Endothelin-1 induces interleukin-6 release via activation of the transcription factor NF-kappaB in human vascular smooth muscle cells. Basic Res. Cardiol..

[88] Verma S., Li S.H., Badiwala M.V., Weisel R.D., Fedak P.W., Li R.K., Dhillon B., Mickle D.A. (2002). Endothelin antagonism and interleukin-6 inhibition attenuate the proatherogenic effects of C-reactive protein. Circulation.

[89] Ho C.H., Wu C.C., Wu C.C., Tsai Y.C. (2019). Laparoscopic total extraperitoneal inguinal hernia repair is safe and feasible in patients with continuation of antithrombotics. J. Minim. Access Surg..

[90] Aldiss P., Davies G., Woods R., Budge H., Sacks H.S., Symonds M.E. (2017). ‘Browning’ the cardiac and peri-vascular adipose tissues to modulate cardiovascular risk. Int. J. Cardiol..

