# Supplementary material for: Ecklonia Cava Extract Attenuates Endothelial Cell Dysfunction by Modulation of Inflammation and Brown Adipocyte Function in Perivascular Fat Tissue
Source: Nutrients. 2019 Nov 15;11(11):2795. doi: 10.3390/nu11112795 (PMC6893767; doi:10.3390/nu11112795)
Supplement: Supplementary file 1 [file nutrients-11-02795-s001.pdf]

Supplementary table 1. List of primer for qRT-PCR

Gene		Primers
β-actin	Forward	5'-ACAAAGCTGTTCAGTGTCTCCA-3′
	Reverse	5'-CTCCGTTTCCAGAATACACACA-3′
iNOS	Forward	5'-CACAGCAATATAGGCTCATCCA-3′
	Reverse	5'-AGCCTCATGGTAAACACGTTCT-3′
CD80	Forward	5'-GACCGAATCTACTGGCAAAAAC-3′
	Reverse	5'-TTCTTATACTCGGGCCACACTT-3′
Arg-1	Forward	5'-ACAGAACTAAGCAAACGCCTTC-3′
	Reverse	5'-AGAAAGGAACTGCTGGGATACA-3′
CD206	Forward	5'-TGTATTCTTTGCCTTTCCCAGT-3′
	Reverse	5'-GATAAAAGCCAGAAGCAGGAGA-3′
MCP-1	Forward	5'-AGGTGTCCCAAAGAAGCTGTAG-3′
	Reverse	5'-AATGTATGTCTGGACCCATTCC-3′
TNF-α	Forward	5'-TTCTGTCTACTGAACTTCGGGGTGATCGGTCC-3′
	Reverse	5'-GTATGAGATAGCAAATCGGCTGACGGTGTGGG-3′
IL-6	Forward	5'-ACTGGGGATGTCTGTAGCTCAT-3′
	Reverse	5'-GGGAGTGGTATCCTCTGTGAAG-3′
IL-10	Forward	5'-ATGGTGTCCTTTCAATTGCTCT-3′
	Reverse	5'-AGGATCTCCCTGGTTTCTCTTC-3′
Chemerin	Forward	5'-TGATCTACAGCTTGGTGTGCTT-3′
	Reverse	5'-TTCACGGTCTTCTTCATCTTGA-3′
IRE-1a	Forward	5'-ATCTGAAAAGGTTCCGCTCAT A-3′
	Reverse	5'-TAGTGGTGCTTCTTGTTCCTCA-3′
Xbp-1	Forward	5'-TTGAGGAAGCACCTCTAAGCT C-3′
	Reverse	5'-GGATGAAGTCATCTTCCAAAG G-3′
PERK	Forward	5'-CATCAGCACTTTAGATGGACGA-3′
	Reverse	5'-AGATGAAACCAAGGAACCAGAC-3′
eIF1a	Forward	5'-TATGCTCAGGTGATCAAAATGC-3′
	Reverse	5'-GCTTCCCTCTTATATGGCACAG-3′
CHOP	Forward	5'-GCTCTCCAGATTCCAGTCAGAG-3′
	Reverse	5'-ACCACTCTGTTTCCGTTTCCTA-3′
PRDM16	Forward	5'-TTGAGGATATCAACACCACGAC-3′
	Reverse	5'-TTGCCTTTGTCTCTGTCACTGT-3′
PPARγ	Forward	5'-TGGCAAAGCATTTGTATGACTC-3′
	Reverse	5'-ATTTGTCCGTTGTCTTTCCTGT-3′
CIDEA	Forward	5'-GAAAAGGGACAGAAATGGACAC-3′
	Reverse	5'-GCCTGTATAGGTCGAAGGTGAC-3′
Eva1	Forward	5'-AGGTGAAGAATCCACCTGATGT-3′

	Reverse	5'-AGATCTCAGAGAAGGGCACAGT-3′
Ebf3	Forward	5'-GCTGTGACAAGAAGAGTTGTGG-3′
	Reverse	5'-TCTGATTGCACTTGAGGAAGAA-3′
Hsbp7	Forward	5'-GTTTTCAGAGGACTTTGGCAGT-3′
	Reverse	5'-CCGAGAGTCTTGATGTTTCCTT-3′
ZIC1	Forward	5'-CCCATCAAGCAAGAGCTTATCT-3′
	Reverse	5'-CTGAAAGTTTTGTTGCACGACT-3′

Supplementary figure 1. Summarized scheme images for experiments

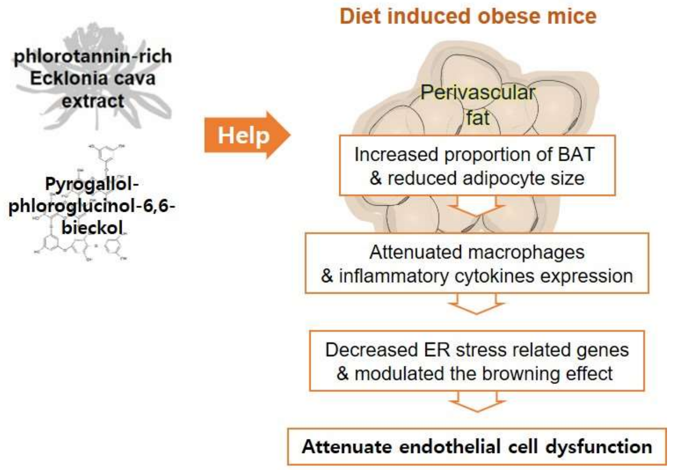

ECE and PPB increased the proportion of BAT and reduced adipocyte size in diet-induced obese mice (DIO). Both attenuated DIO-induced increases and decreases in M1 and M2 macrophages and inflammation in PVAT, respectively and they also attenuated ER stress and modulated the browning effect in the PVAT. Finally ECE and PPB attenuate endothelial cell dysfunction in DIO mice.
